# Validation of verbal autopsy and nasopharyngeal swab collection for the investigation of deaths at home during the COVID-19 pandemics in Brazil

**DOI:** 10.1371/journal.pntd.0008830

**Published:** 2020-11-04

**Authors:** Pedro Mansueto Melo de Souza, Gunter Gerson, Josebson Silva Dias, Deborah Nunes de Melo, Sarlene Gomes de Souza, Erasmo Miessa Ruiz, Fabio Rocha Fernandes Tavora, Luciano Pamplona de Góes Cavalcanti

**Affiliations:** 1 Serviço de Verificação de Óbitos Dr. Rocha Furtado, Fortaleza, Ceará, Brasil; 2 Universidade Estadual do Ceará, Fortaleza, Ceará, Brasil; 3 Universidade Federal do Ceará, Fortaleza, Ceará, Brasil; 4 Brown University, Providence, Rhode Island, United States of America; 5 Centro Universitário Christus, Fortaleza, Ceará, Brasil; Institute of Tropical Medicine Antwerp, BELGIUM

Brazil currently holds the second highest record of cases and deaths due to the Coronavirus Disease 2019 (COVID-19) in the world [1]. The state of Ceará, with >9 million inhabitants, in Northeastern Brazil, was the epicenter of the epidemic and one of the first states to present community transmission of the COVID-19. Forty-five days after the first confirmed case, there was a collapse of health services, contributing to the increase in the number of people dying at home [2].

In the scenario of a pandemic associated with an increase in the number of deaths outside a hospital environment, a specialized service in the investigation of fatalities was necessary to increase the capacity of the health system and to detect and report the death burden from the disease. “Dr. Rocha Furtado” Death Verification Service (SVO-RF) is located in the capital Fortaleza and, in partnership with the Epidemiological Surveillance Office and the local public health laboratory, it acts in the investigation of deaths in the state, which was already reported in recent arboviruses epidemics [3,4].

To fulfill its mission, SVO-RF performs complete diagnostic autopsies (CDA) for the investigation of deaths at home without medical assistance, as well as deaths without bona fide diagnosis prior to death [5]. Moreover, unlike most other Death Verification Services in Brazil, the SVO-RF also implemented a medical team that drives to the houses where the death occurred, to investigate cases where a clinical necropsy was not indicated, such as with patients with advanced cancer diagnoses or other chronic terminal illnesses that die at home, without any home care program. This team, called SVO-Mobile, is composed of a physician, a social worker, and a driver and usually acts in a complementary way to clinical necropsies that take place at the headquarters. In the COVID-19 pandemic, however, the SVO-Mobile played a significant role since clinical necropsies were suspended following the Ministry of Health’s guidelines [6].

Due to the lack of biosafety at the headquarters, including an air treatment system at the facility, added to the community transmission of the virus and the risk of transmission by asymptomatic and presymptomatic patients, all autopsies were suspended. The SVO-RF leadership saw then the need to expand the number of SVO-Mobile teams from 1 to 3, while still maintaining its regular team at the headquarters. All deaths from this period were investigated through physician-certified verbal autopsies (PCVA), external body examination, and collection of nasopharyngeal swab samples in cases of suspected COVID-19, with the 3 SVO-Mobile teams moving to their homes while continuing to receive bodies at headquarters.

From the 10th to the 31st epidemiological week (EW) of 2020, including March 1 to August 1, the number of household deaths in the city of Fortaleza attended by SVO-RF was 2,215, representing an increase of 69.00% to the same period of 2018 and 2019. The weekly percentage variation between the number of consultations in 2020 with the average of 2018 to 2019 varied from −14.56% in the 30th EW to +355.65% in the 19th EW (Table 1).

**Table 1:** Deaths at home in Fortaleza, State of Ceará, attended by SVO-RF in 2018, 2019, and 2020, suspicious and confirmed home deaths by COVID-19 in 2020.

EW	N of home deaths in 2018	N of home deaths in 2019	N of home deaths in 2020	% change in the number of deaths (2020/average 2018–2019)	N of suspected COVID-19 deaths in 2020	% of suspected COVID-19 deaths in 2020	N of deaths by COVID-19 confirmed in 2020	% of deaths by COVID-19 confirmed in relation to suspects in 2020
10	63	56	58	−2.52	0	0.00	0	0.00
11	59	66	57	−8.80	0	0.00	0	0.00
12	62	64	62	−1.59	1	1.61	0	0.00
13	64	66	76	16.92	4	5.26	2	50.00
14	60	49	75	37.61	5	6.67	1	20.00
15	53	66	82	37.82	9	10.98	5	55.56
16	53	52	103	96.19	14	13.59	7	50.00
17	72	70	96	35.21	15	15.63	13	86.67
18	59	60	169	184.03	39	23.08	39	100.00
19	46	69	262	355.65	86	32.82	78	90.70
20	77	66	236	230.07	89	37.71	75	84.27
21	55	66	173	185.95	41	23.70	37	90.24
22	47	45	118	156.52	20	16.95	15	75.00
23	62	53	85	47.83	8	9.41	3	37.50
24	38	53	75	64.84	6	8.00	2	33.33
25	42	48	64	42.22	3	4.69	2	66.67
26	56	60	50	−13.79	4	8.00	1	25.00
27	62	43	57	8.57	3	5.26	0	0.00
28	68	49	58	−0.85	1	1.72	0	0.00
29	60	46	63	18.87	2	3.17	0	0.00
30	58	45	44	−14.56	2	4.55	0	0.00
31	52	43	52	9.47	1	1.92	0	0.00
Total	**1,268**	**1,235**	**2,115**	**69.00**	**353**	**16.69**	**280**	**79.32**

EW, epidemiological week; N, number.

Among the 2,115 household deaths in this period, 353 (16.69%) cases had clinical–epidemiological criteria for suspected COVID-19, with the weekly variation starting from 0.00% in the first 2 weeks studied, up to 37.71% in the 20th EW (Table 1).

For any suspected case, nasopharyngeal swab samples were collected and sent to the Central Laboratory of Public Health of Ceará for the Severe Acute Respiratory Syndrome Coronavirus 2 (SARS-CoV-2) virus polymerase chain reaction technique in real-time (RT-PCR) testing. Among the suspected cases, the rate of positivity in the SARS-CoV-2 survey ranged from 0.00% at the beginning and end of the period analyzed to 100.00% positivity in the 18th EW (Table 1). It is a noteworthy fact that there were no confirmed cases of SARS-CoV-2 among home deaths in the last 5 weeks, which may represent both a decrease in the circulation of the virus as well as the reestablishment of the capacity of the health services of providing medical assistance to possible new cases of the disease.

Therefore, in the light of an increase of up to 355.65% in the weekly number of home deaths, it was the ability to rapidly expand the investigation teams that made it possible to meet the excessive demand of these 2,115 deaths that occurred outside the hospital environment and confirm 280 deaths from COVID-19 that would hardly have been identified without this service (Table 1). Nevertheless, the use of the verbal autopsy did not allow the medical team to answer some questions about other home deaths that did not meet the criteria for suspicion for COVID-19. For example, would it be possible that all the excess of unsuspected deaths from COVID-19 resulted only from the difficulty of accessing health services? Or could some of these deaths have been caused by COVID-19 with an atypical clinical presentation without criteria for suspicion?

In view of these limitations, we believe it is necessary to invest also in other forms of investigation, such as minimally invasive necropsies, whose applicability and safety have already been demonstrated in other developing countries [7], with infectious diseases in general [8] and with COVID-19 specifically [9]. We also advocate the expansion of biosafety measures for the entire network of Death Verification Services, so that complete clinical necropsies can be performed even during pandemics, following the recommended good laboratory practices [10] and contributing to the understanding of the pathophysiological mechanisms that lead to death by COVID-19 [11] worldwide.

Despite these and other limitations of the verbal autopsy certified by a physician [12], we consider that, in a pandemic scenario, the investigation of home deaths by this method associated with the collection of samples for laboratory research constitutes a safe, financially viable, and secure method. It probably contributes to an increase in the detection of the disease and a consequent decrease in underreporting, especially in places where the collapse of the health system can lead to a rise in home deaths without medical assistance.
